# The impact of the affordable care Act (ACA) on favorable risk selection and Beneficiaries’ health status in Medicare advantage: a preliminary assessment

**DOI:** 10.1186/s12913-016-1663-4

**Published:** 2016-08-18

**Authors:** Nabil Natafgi, Matthew Nattinger, Patience Ugwi, Fred Ullrich, Fredric D. Wolinsky

**Affiliations:** 1 Department of Health Management and Policy, College of Public Health University of Iowa, 145 River Side Drive, Iowa City, IA 52241 USA; 2 Stead Family Department of Pediatrics, University of Iowa Carver College of Medicine, University of Iowa, 200 Hawkins Drive, Iowa City, IA 52242 USA

**Keywords:** Affordable Care Act (ACA), Healthcare Reform, Medicare Advantage, Medicare Payment, Favorable Risk Selection, Health Outcomes

## Abstract

**Background:**

In response to increasing fiscal pressures, the Affordable Care Act (ACA) sought to reduce Medicare Advantage plan expenses by restructuring the bidding and payment processes. The purpose of this study is to assess the effects of the ACA’s payment freeze and restructuring of the bidding and payment processes on favorable risk selection in Medicare Advantage plan enrollment (objective 1) and changes in the health status of beneficiaries enrolled in Medicare Advantage plans over time (objective 2).

**Methods:**

We used the Medicare Health Outcome Survey baseline data (2007→2013) for analyses of the first objective (7 cohorts, 1.7 million beneficiaries) and the linked baseline and follow-up data (2007–2009→2011–2013) for analyses of the second objective (5 cohorts, 0.5 million beneficiaries). To examine favorable risk selection we used the following outcomes: self-rated health, falls, balance problems, falls management, frailty, and morbidity. To examine changes in beneficiary health status over time, we examined changes (over time) in these same outcomes. The focal independent variable is the policy implementation measure, which is time dependent and measures the accumulation of changes to Medicare Advantage payment policies resulting from the ACA. Multiple regression models were developed to examine the relationship between ACA implementation and outcomes of interest.

**Results:**

In terms of favorable selection, individuals enrolled in Medicare Advantage plans post-ACA have, on average, better self-rated health (b = 0.003, *p* < 0.01), lower odds of falls (AOR = 0.981, *p* < 0.001), higher odds of falls management (AOR = 1.040, *p* < 0.001), lower frailty risks (IRR = 0.983, *p* < 0.001), and lower risks of comorbidities (IRR = 0.989, *p* < 0.001). In terms of health status changes over time, the results indicate that in the post-ACA period, beneficiaries reported better self-rated health (b = 0.028, *p* < 0.001), lower odds of falls (AOR = 0.965, *p* < 0.001), lower odds of balance problems (AOR = 0.958, *p* < 0.001), lower odds of falls management (AOR = 0.981, *p* < 0.05), lower frailty risks (IRR = 0.944, *p* < 0.001), and lower risks of comorbidity (IRR = 0.986, *p* < 0.001) at follow up compared to the same risks at baseline.

**Conclusions:**

These findings suggest that as the Medicare Advantage payment policies in the ACA were being implemented, plans may have engaged in favorable selection activities, yet beneficiaries exhibited more favorable health outcomes.

## Background

Medicare Advantage (MA) - offered by private plans - is a market-based managed care alternative to the traditional fee-for-service (FFS) model that provides Medicare beneficiaries with both Part A (inpatient services) and Part B (outpatient services) benefits. Through contracted arrangements with the Centers for Medicare and Medicaid Services (CMS), the private managed care plans are paid a preset per member per month (capitation) payment according to certain benchmarks following a bidding process that determines the maximum amount Medicare will pay.

In 2003, the Medicare Prescription Drug, Improvement and Modernization Act (MMA) increased federal payments to MA plans to expand their role under Medicare and offer beneficiaries a wider choice of plans as well as access to extra benefits. By 2010, this led to an increase in the ratio of per-capita payments for MA beneficiaries compared to the cost of care under the traditional FFS Medicare (113 % of average per-capita costs under FFS Medicare), and greater MA market penetration (25 %) [1]. The cost of these higher payments for an expanding proportion of beneficiaries was one of the core factors threatening Medicare’s fiscal sustainability.

In response to these fiscal pressures and to partially cover the added costs associated with expanding health insurance coverage to millions of previously uninsured Americans, the Patient Protection and Affordable Care Act (ACA) of 2010 set about to reduce MA plan payments using three distinct policy levers. The first was freezing MA 2011 payments at their 2010 levels [2]. The second was phasing-in restructured bidding and payment processes that used blended benchmarks set at increasingly lower percentages of FFS Medicare rates. Blending now primarily reflects county rather than national costs, with benchmarks set at 95 % for counties in the highest FFS spending quartile, and 100, 107.5, and 115 % for counties in the progressively lower spending quartiles [2]. When fully implemented it is estimated this will result in a 13 % reduction or about $1,538 per-capita in MA payments, with substantial variation in reduction amounts by county [3, 4]. These policies are planned to be phased-in over 2–6 years 2011–2017) depending on the amount of the plan’s anticipated payment reduction, with earlier phase-ins for lower reductions and longer phase-ins for higher reductions [5].

The third lever was the linkage of rebate and bonus payments to the quality of care that plans provided. The quality scores for MA plans are based on a set of performance measures assessing plans’ processes, management of chronic conditions, beneficiaries’ health, and member experience and customer service [6, 7]. MA plans are rated on a 1 to 5 star scale, with 1 star representing poor performance, and 5 stars representing excellent performance. Rebate rates range from 70 % for plans with 4.5 or 5 Medicare quality stars, to 50 % for those with 3 or fewer quality stars [5]. Plans with 3 or more Medicare quality stars will get a 5 % bonus with 3 % going to lower quality plans.

MA plans have always had the incentive to engage in favorable risk selection practices by targeting healthier, lower-cost beneficiaries to encourage them to enroll in MA. Favorable selection means beneficiaries who cost less-than-average disproportionately enroll in MA, while those who cost more-than-average disproportionately remain in traditional FFS Medicare [8]. The MA payment changes brought about by the ACA may have stimulated plans to intensify their favorable risk selection efforts, improve their Medicare quality star ratings, reduce or constrain the health services they provide, or raise their bids above the benchmark, which may have led to changes in beneficiary health outcomes.

Several studies have examined the potential effects of these and other policy changes on MA-to-FFS and FFS-to-MA switching, plan selection, plan availability, plan quality star ratings, race/ethnic plan composition, and favorable selection [2–4, 8–19]. These studies reflect varying conclusions and trends, including reductions in the number of available plans [10], reductions in the ratio of MA plan payments to average per-capita costs under FFS Medicare from 14 to 9 % [4], declines in favorable selection [8, 9, 11], increased dis-enrollment rates among dual-eligible and disability-eligible beneficiaries [7], modest changes in enrollee risk scores [15], increased member monthly premiums among higher quality plans that were also more likely to drop their $0 premium plans [13], no significant reductions in health services provided or increases in beneficiary costs [14], higher switching rates from MA to FFS than from FFS to MA [16], diminished returns to plan quality on beneficiary plan choice at higher plan quality levels with plan choice influenced primarily by plan market share [17], little evidence of MA plans under-enrolling race and ethnic minorities [18], and higher quality ratings linked to nonprofit status and plan endurance (age) [19].

While these studies have examined the effects of ACA-induced MA policy changes on different dimensions, the majority rely on only 1–2 years of data, none have used data more recent than 2011, and none have focused on beneficiary health outcomes. To that end, this paper aims to focus on the effects at the beneficiary level of the ACA’s payment freeze and newly blended benchmarks on favorable risk selection in enrollments (*objective 1*) and changes in the health status of beneficiaries enrolled in MA plans (*objective 2*) over time.

## Methods

### Data sources and study population

We used the Medicare Health Outcome Survey (HOS) developed by CMS for all analyses. The HOS is an annual patient-reported survey dataset compiled from a nationally representative random sample of beneficiaries enrolled in MA plans with at least 500 members. It is used by CMS to monitor and evaluate the quality of health care provided to MA plan beneficiaries and to set MA plan payment, rebate, and bonus levels. The survey is administered to a new baseline cohort of MA plan beneficiaries every year who are then resurveyed for follow-up after 2 years. The survey protocol includes four main components: (i) the Veterans RAND 12-Item Health Survey (VR-12); (ii) case-mix and risk-adjustment questions; (iii) questions that tap on HEDIS Effectiveness of Care measures; and (iv) additional health questions (e.g., cognitive function, memory, living arrangements) [20].

The analytic sample was limited to individuals who were age-eligible for Medicare (aged 65 years old or older). We used seven consecutive HOS cohorts: Cohort 10 (2007–2009) to Cohort 16 (2013–2015). Specifically, we used only baseline data (2007→2013) for the analyses of the first objective (7 cohorts and 1.7 million beneficiaries) and the linked baseline and follow-up data (2007–2009→2011–2013) for the analyses of the second objective (5 cohorts and 0.5 million beneficiaries; follow-up data for Cohorts 15 and 16 have not yet been released at the time of data analysis).

### Dependent variables

To examine favorable risk selection into MA plans we used the following outcomes at baseline: self-rated health (SRH), falls, balance problems, falls management, frailty, and morbidity. And to examine changes in beneficiary health status over time, we looked at changes in these same outcomes after 2 years. SRH is the traditional self-assessment of beneficiaries’ perceived health status, in which beneficiaries were asked to respond to the question “In general, would you say your health is:” and the response items were “Excellent”, “Very Good”, “Good”, “Fair”, “Poor”. The SRH variable was coded from 1 “Poor” to 5 “Excellent”.

Falls is a binary variable for falls in the previous year, in which beneficiaries were asked to respond to the question “Did you fall in the past 12 months?” and is coded 1 for “Yes”. Balance problems is a binary indicator for problems with balance or walking, in which beneficiaries were asked to respond to the question “In the past 12 months, have you had a problem with balance or walking?” and is coded 1 for “Yes”. Falls management is a binary indicator for discussion with a doctor about how to prevent falls, in which beneficiaries were asked to respond to the question “A fall is when your body goes to the ground without being pushed. In the past 12 months, did you talk with your doctor or other health provider about falling or problems with balance or walking?” and coded 1 for “Yes”.

Frailty is a count variable reflecting the number of impairments in the activities of daily living (ADLs): bathing, dressing, eating, getting in or out of chairs, walking, and using the toilet. Beneficiaries’ were asked to respond to the following question “Because of a health or physical problem, do you have any difficulty doing the following activities without special equipment or help from another person?”. Individual ADL item responses were first transformed to binary indicators coded 1 for “Yes, I have difficulty” or “I am unable to do this activity” and 0 for “No, I do not have difficulty”. The Frailty variable is the numeric sum of the 6 individual indicators of ADL.

Morbidity is a count variable of the number of morbidities reported by beneficiaries in response to “Has a doctor ever told you that you had:”. Responses were binary (Yes/No) for each of the following 13 health conditions: hypertension, angina, congestive heart failure, myocardial infraction, other heart condition, stroke, emphysema, inflammatory bowel disease, arthritis of hip or knee, arthritis of hand or wrist, sciatica, diabetes, any cancer. The morbidity score is the numeric sum of the 13 individual indicators of health condition truncated at 7 comorbidities (i.e., individuals with 8 conditions or more [3 % of the sample size] were assigned to ‘7’). Similar weight was applied to all activities of daily living and chronic conditions in the calculations of ADL and morbidity scores.

### Independent variables

The focal independent variable in this study is the policy implementation measure (PIM), which is time-dependent and measures the accumulation of changes to MA payment policies resulting from the ACA. PIM is set at ‘0’ for each of the pre-ACA years (2007–2010), ‘1’ for 2011 to reflect the freeze in MA plan payments at their 2010 levels, ‘2’ for 2012 to account for the original payment freeze and the restructuring of the bidding and payment processes (first set of county-based cuts), and ‘4’ for 2013 to account for the original payment freeze, the first and second set of county-based cuts, and sequestration (which began in 2013 as part of the 2011 Budget Control Act) [21]. We included a time-dependent secular trend measure coded ‘1’ for 2007 through ‘7’ for 2013 to adjust for temporal changes in the outcomes of interest between 2007 and 2013. We also adjusted for age, sex, race-ethnicity, education, marital status, and health status.

### Data analysis

#### Objective 1

To assess whether the payment changes led to favorable risk selection in MA plan enrollment, we examined changes over time in health characteristics using baseline data from 2007 to 2013. The model is expressed as:

1 $$ {Y}_1=a + {\beta}_1PIM + {\beta}_2 Trend+{\beta}_3Dem+{\beta}_4 Health+e $$

where *Y*_1_ is the health outcome at baseline (either SRH, falls, balance problems, falls management, frailty, or morbidity), *PIM* is the policy implementation measure, *Trend* is the secular trend indicator, *Dem* includes patient demographic characteristics (i.e., age, sex, race-ethnicity, education, marital status), and *Health* includes health status measures other than the outcome of interest. We introduced patient demographics and health status variables (*Dem* and *Health*) successively in a 3-step fashion (stepwise regression). The first model includes the two focal variables of interest (i.e., secular trend and policy effect). The second model includes patient demographics (age, sex, race-ethnicity, education, and marital status) in addition to the two focal variables of interest. The third model is the complete (and preferred) model and adjusts for health status of the individuals (SRH, falls, balance problems, falls management, frailty, and morbidity) in addition to their demographics. A health status adjustor variable is not included when it is the dependent variable. For quasi-interval outcomes (SRH) we use ordinary least squares (OLS) regression, for count outcomes (frailty and morbidity) we use negative binomial regression models, and for binary outcomes (falls, balance problems, and falls management) we use logistic regression models [22, 23].

#### Objective 2

To assess whether the payment changes induced by the ACA led to changes in beneficiaries’ health status over time, we examined temporal changes in the health status of MA beneficiaries. The full model is expressed as:

2 $$ {Y}_2=a + {\beta}_0{Y}_{baseline}+{\beta}_1PIM+{\beta}_2 Trend+{\beta}_3Dem+{\beta}_4 Health+e $$

where *Y*_*2*_ is the follow-up value for the outcome of interest (either SRH, falls, balance problems, falls management, frailty, or morbidity). The predictor variables are the same as used in the first objective, except that the baseline value of the outcome measure of interest (*Y*_*baseline*_) is included. For quasi-interval (SRH) outcomes we use ordinary least squares (OLS) regression, for count outcomes (frailty and morbidity) we use negative binomial regression models, and for binary outcomes (falls, balance problems, and falls management) we use logistic regression models [22, 23]. Data were analyzed with SPSS Statistics version 22.0 software (IBM SPSS, Inc, NY).

## Results

Table 1 summarizes the demographic characteristics and health status of the MA beneficiaries included in the analytic sample for each of the two objectives. At baseline for objective 1, more than half of the beneficiaries were females (58.5 %), married (54.2 %), or between 65 and 74 years old (58.2 %). The majority were white (81.1 %) and more than one-third had greater than a high school education (38.8 %). In terms of health status, on average, 30.3 % rated their health as below average (fair or poor), 23.0 % fell in the previous 12 months, one-third experienced problems in balance or walking (33.5 %) and 30.7 % talked to the doctor about fall prevention. On average, the MA enrollees had a single disability (ADL score of 0.99) and three comorbidities (morbidity score of 2.86). The characteristics of objective 2 population at baseline were very similar to those for objective 1.

**Table 1:** Descriptive Statistics

Variable	N (%)		
	Objective 1	Objective 2 at Baseline	Objective 2 at Follow up
	*n* = 1,658,453	*n* = 546,362	*n* = 546,362
Age			
65 to 74	964,956 (58.2)	324,065 (59.3)	
75 and older	693,497 (41.8)	222,297 (40.7)	
Gender			
Female	938,226 (58.5)	318,329 (58.9)	
Male	666,270 (41.5)	221,773 (41.1)	
Race			
White	1,190,929 (81.1)	440,198 (83.3)	
Black or African American	144,437 (9.8)	42,722 (8.1)	
Other	132,635 (9.0)	45,814 (8.7)	
Marital Status			
Married	816,103 (54.2)	306,183 (56.9)	
Non-married	688,321 (45.8)	231,489 (43.1)	
Education			
Less than high school education or equivalent	397,151 (26.6)	130,480 (24.4)	
High school education or equivalent	518,306 (34.7)	194,584 (36.3)	
Greater than high school education or equivalent	579,193 (38.8)	210,315 (39.3)	
Self-Rated Health (SRH)			
Excellent	102,591 (6.5)	35,892 (6.7)	30,871 (5.7)
Very good	393,205 (25.0)	148,911 (27.6)	138,340 (26.3)
Good	599,089 (38.2)	216,901 (40.3)	208,904 (39.7)
Fair	370,320 (23.6)	114,631 (21.0)	119,286 (22.6)
Poor	104,673 (6.7)	22,406 (4.1)	29,331 (5.6)
Falls			
Did not fall	1,163,694 (77.0)	428,294 (78.4)	400,071 (77.3)
Fell to the ground	347,670 (23.0)	110,755 (20.5)	117,289 (22.7)
Balance Problems			
No	1,002,304 (66.5)	379,692 (70.5)	338,669 (65.7)
Yes	505,307 (33.5)	158,532 (29.5)	177,149 (34.3)
Falls Management			
No	1,019,652 (69.3)	389,581 (74.1)	348,958 (69.2)
Yes	451,062 (30.7)	135,948 (25.9)	155,283 (30.8)
Variable (range)	Mean (SD)		
	Objective 1	Objective 2 at Baseline	Objective 2 at Follow up
Frailty - ADL Score^a^ (0–6)	0.99 (1.66)	0.79 (1.44)	0.95 (1.60)
Morbidity Score^b^ (0–7)	2.86 (1.93)	2.76 (1.88)	2.88 (1.90)

^a^Activities of daily living (ADL) score calculated as the sum of the six disability components (binary indicators): bathing, dressing, eating, getting in or out of chair, walking and using the toilet

^b^Morbidity score is the sum of 13 comorbidity dummy indicators truncated at 7 comorbidities

Unadjusted trends of health status measures at baseline showed that between 2007 and 2009, beneficiaries showed a decreasing trend in SRH that was reversed in 2010. In addition, falls and balance problems among beneficiaries at baseline were increasing prior to 2010, but this trend was halted in 2010. Frailty and morbidity among beneficiaries at baseline was increasing prior to 2010, but decreased after the changes in MA reimbursement policies began.

### Objective 1

Table 2 shows the results of the regression models assessing favorable risk selection in the enrollment of beneficiaries in MA plans. The first model shows the unadjusted effects of the policy implementation and secular trend variables. The second and third models successively adjust for demographics and health status. The results indicate that after adjustment for demographics and health status, individuals enrolled in MA plans post-2010 (the year in which changes in MA reimbursement started to take effect) have, on average, better SRH (b = 0.003, *p* < 0.01), lower odds of falls (AOR = 0.981, *p* < 0.001), higher odds of falls management (AOR = 1.040, *p* < 0.001), lower frailty risks (IRR = 0.983, *p* < 0.001), and lower risks of comorbidities (IRR = 0.989, *p* < 0.001).

**Table 2:** First Objective - Favorable risk selection in enrollment

Dependent Variable	Focal Variable^a^	Model 1^b^	Model 2^c^	Model 3^d^
		Effect^e^ (SE)	Effect^e^ (SE)	Effect^e^ (SE)
Self-Rated Health (SRH)	Secular Trend	0.002 (0.001)	−0.005 (0.001)^***^	0.008 (0.001)^***^
	Policy Measure	0.020 (0.001)^***^	0.014 (0.001)^***^	0.003 (0.001)^**^
Falls	Secular Trend	1.020 (0.002)^***^	1.025 (0.002)^***^	1.003 (0.003)
	Policy Measure	0.976 (0.003)^***^	0.975 (0.003)^***^	0.981 (0.003)^***^
Balance Problems	Secular Trend	1.022 (0.002)^***^	1.032 (0.002)^***^	0.994 (0.003)^*^
	Policy Measure	0.984 (0.003)^***^	0.986 (0.003)^***^	1.006 (0.004)
Falls Management	Secular Trend	1.043 (0.002)^***^	1.057 (0.002)^***^	1.044 (0.003)^***^
	Policy Measure	1.006 (0.003)^*^	1.009 (0.003)^**^	1.040 (0.003)^***^
Frailty/disability	Secular Trend	1.025 (0.001)^***^	1.033 (0.001)^***^	1.013 (0.002)^***^
	Policy Measure	0.969 (0.002)^***^	0.973 (0.002)^***^	0.983 (0.002)^***^
Morbidity Score	Secular Trend	1.011 (0.001)^***^	1.013 (0.001)^***^	1.008 (0.001)^***^
	Policy Measure	0.983 (0.002)^***^	0.985 (0.002)^***^	0.989 (0.002)^***^

Significance level: * *p*-value <0.05; ** *p*-value <0.01; *** *p*-value <0.001. Due to space limitations, only focal variables of interest were displayed. Complete tables including coefficients of all included variables are available upon request

^a^Secular Trend is a time-dependent indicator that captures any temporal changes in the outcomes of interest over time between 2007 and 2013; Policy Measure is the policy implementation measure (PIM) which reflects the changes in MA reimbursement

^b^Model 1 includes (i) secular trend variable and (ii) policy effect

^c^Model 2 includes (i), (ii), and (iii) demographics (age, sex, race-ethnicity, education, and marital status)

^d^Model 3 includes (i), (ii), (iii), and (iv) health status (SRH, falls, balance problems, falls management, frailty [when Balance is the DV, ADL score is calculated excluding walking disability component], and morbidity). A health status adjustor variable is not included when it is the DV

^e^Beta Coefficient Effect for OLS predicting SRH; Adjusted Odds Ratio [OR = Exp (B)] predicting falls, balance problems and falls management; Incidence Risk Ratio [IRR = Exp (B)] for Negative Binomial Regression predicting frailty and morbidity

### Objective 2

Table 3 shows the results of the models assessing the changes in health status over time associated with the MA reimbursement reform introduced in 2010. The results indicate that post reimbursement changes, beneficiaries, on average, reported statistically higher SRH (b = 0.028, *p* < 0.001), lower odds of falls (AOR = 0.965, *p* < 0.001) and balance problems (AOR = 0.958, *p* < 0.001), lower odds of falls management (AOR = 0.981, *p* < 0.05), and lower frailty (IRR = 0.944, *p* < 0.001) and fewer comorbidities (IRR = 0.986, *p* < 0.001) at follow-up compared to baseline.

**Table 3:** Second Objective - Changes in health status

Dependent Variable	Focal Variable^a^	Model 1^b^	Model 2^c^	Model 3^d^
		Effect (SE)	Effect (SE)	Effect (SE)
Self-Rated Health (SRH)	Secular Trend	−0.022 (0.002)^***^	−0.019 (0.002)^***^	−0.015 (0.002)^***^
	Policy Measure	0.034 (0.002)^***^	0.028 (0.002)^***^	0.028 (0.002)^***^
Falls	Secular Trend	1.034 (0.009)^***^	1.029 (0.009)^**^	1.020 (0.009)^*^
	Policy Measure	0.958 (0.008)^***^	0.965 (0.008)^***^	0.965 (0.008)^***^
Balance Problems	Secular Trend	1.064 (0.009)^***^	1.055 (0.009)^***^	1.049 (0.009)^***^
	Policy Measure	0.944 (0.008)^***^	0.958 (0.008)^***^	0.958 (0.008)^***^
Falls Management	Secular Trend	1.093 (0.008)^***^	1.083 (0.008)^***^	1.073 (0.009)^***^
	Policy Measure	0.951 (0.008)^***^	0.971 (0.008)^***^	0.981 (0.008)^*^
Frailty/disability	Secular Trend	1.056 (0.006)^***^	1.049 (0.006)^***^	1.043 (0.006)^***^
	Policy Measure	0.930 (0.005)^***^	0.943 (0.005)^***^	0.944 (0.005)^***^
Morbidity Score	Secular Trend	1.005 (0.004)	1.004 (0.004)	1.005 (0.004)
	Policy Measure	0.984 (0.004)^***^	0.986 (0.004)^***^	0.986 (0.004)^***^

Significance level: * *p*-value <0.05 **; *p*-value <0.01; *** *p*-value <0.001. Due to space limitations, only focal variables of interest were displayed. Complete tables including coefficients of all included variables are available upon request

^a^Secular Trend is a time-dependent indicator that captures any temporal changes in the outcomes of interest over time between 2007 and 2013; Policy Measure is the policy implementation measure (PIM) which reflects the changes in MA reimbursement

^b^Model 1 includes (i) secular trend variable, (ii) policy effect, (iii) outcome indicator at baseline

^c^Model 2 includes (i), (ii), (iii), and (iv) demographics (age, sex, race-ethnicity, education, and marital status)

^d^Model 3 includes (i), (ii), (iii), and (iv) health status (SRH, falls, balance problems, falls management, frailty [when Balance is the DV, ADL score is calculated excluding walking disability component], and morbidity). A health status adjustor variable is not included when it is the DV

## Discussion

There is limited information about the effects of the ACA on MA plan behavior and beneficiary health. This study is important because it fills gaps in the literature and provides policymakers with critical information that is needed to shape policy development. This work provides preliminary evidence on favorable selection in MA enrollment and MA beneficiary health over time in response to ACA changes in MA payment policies.

The results indicate that, as the ACA is being implemented, MA plans are seeking to recruit healthier seniors during enrollment suggesting intensified efforts at favorable risk selection. While this is not surprising [1, 11, 24, 25], it is an important finding because it signals a reverse in the recent success of Medicare efforts to reduce favorable risk selection in MA plans by revising the risk adjustment formula and restricting monthly disenrollment by beneficiaries [8, 9].

Based on earlier evidence, some studies anticipated a decrease in enrollment of new beneficiaries in MA plans associated with a substantial period of disenrollment when current enrollees return to FFS Medicare [8]. Such predictions appear to be only partially correct. Recent updates on MA enrollment show an increase in the number and share of Medicare beneficiaries in MA plans after the ACA [25]. However, the newly enrolled beneficiaries in MA plans tend to be healthier individuals with less frailty and fewer comorbidities. This is consistent with recent evidence indicating that the switching rate from 2010 to 2011 away from MA and to FFS Medicare exceeded the rate in the opposite direction for participants, particularly among high-cost patients [16].

Another noteworthy finding of this study is the changes in health status of MA beneficiaries associated with the changes in MA payment policy. Despite the importance of health outcomes assessment as efforts to contain health care costs and concerns about the quality of care increases, there is little evidence on health status of MA beneficiaries. A single study compared MAs and the Veterans Health Administration (VHA) with regard to changes in health status and mortality [26]. The authors concluded that there were small differences in 2-year health outcomes that favored the VHA.

Our results indicate that in the post-ACA period, MA enrollees had improved SRH, fewer falls, lower frailty scores, and fewer comorbidities. This could be attributed to MA plans’ increased focus on prevention, coordinated care, and better management of chronic conditions associated with the reimbursement reform efforts that linked MA payments to certain outcome measures. The fixed capitation payment scheme, in addition to the financial rewards under ACA for receiving a higher rating based on performance on measures of quality and patient experience, provides strong incentives to provide more coordinated, effective, and efficient care [4, 27].

Despite these efforts to enhance the quality of care and improve patient outcomes, the findings indicate that in the same post-ACA period MA enrollees had lower odds of receiving an appropriate management of fall risks. One possible explanation may be that the plans are only focusing on the performance measures outlined by CMS under the ACA, which focus on: (i) how often members got various screening tests, vaccines, and other check-ups that help them stay healthy; (ii) how often members with different conditions got certain tests and treatments that help them manage their conditions; (iii) ratings of member satisfaction with the plan [4]. Since it is not one of the explicit measures required for MA plan payment bonuses and given the prevalence of fall risk among Medicare beneficiaries and the multiple providers who would require payment, the plans seems not to be focusing on fall-risk evaluation and management services due to their substantial costs [28].

This study is limited in three important ways. First, the study design cannot tease out other policies that could have affected MA plans post-2010 such as the American Taxpayer Relief Act and the Budget Control Act. Nonetheless, both of those Acts are believed to have had minimal effects on the observed outcomes, as they were only effective as of 2013 and their effect was incorporated in the PIM. The second limitation is the relatively modest longitudinal post-ACA run of the data. Nonetheless, we did observe significant effects in the short-term. Lastly, this study does not account for differences in plan characteristics or the actual changes in MA payments. It is expected that the effects of the PIM will vary by county characteristics, such as market competition and/or penetration rates, as well as plan characteristics. Because these discounts are being phased in over 2–6 years, the first two years available for analysis in this paper reflect the correct proportional changes anticipated in the payment policies. Future research should expand the longitudinal run of the data and focus on changes in the outcomes associated with variations in plan and geographic characteristics.

## Conclusions

This study reflects the first effort to evaluate the effects of the ACA-induced MA payment changes on health outcomes of MA enrollees. The evidence suggests that as the ACA payment policies were being implemented, MA plans engaged in favorable selection activities, yet their beneficiaries exhibited more favorable health outcomes over time.

## Abbreviations

ACA: Patient protection and affordable care act
ADL: Activities of daily living
AOR: Adjusted odds ratio
CMS: Centers for medicare and medicaid services
FFS: Fee-for-service
HOS: Medicare health outcome survey
MA: Medicare advantage
MMA: Medicare prescription drug, improvement and modernization act
PIM: Policy implementation measure
SRH: Self-rated health
VHA: Veteran health administration
